# The Cause and Prevention of Dental Caries

**Published:** 1882-05

**Authors:** W. A. Knowles


### THE CAUSE AND PREVENTION OF DENTAL CARIES.

BY W. A. KNOWLES, M.D., D.D.S.

Dental caries is an inflammation of dentine, terminating in death and disintegration of a part or whole of a tooth.

Caries is generally occasioned by the action of an irritant. It first attacks the organic portion of the tooth, inflammation and death ensue, and this is followed by disintegration of the inorganic portion.

Before decalcification of tooth-tissue can occur, it is absolutely necessary that death of the organic portion should previously have taken place. Vitality resists caries, and in proportion to the life force will this resistance be. Vital force is subject to physiological laws; but as soon as death ensues, chemical laws take charge of the tissue. We find that in a dead tooth, offering no such resistance, disintegration takes place with greater rapidity than in a live tooth under the same conditions.

The first change occurring in dentine after the inception of inflammation is that the contents of the dentinal tubes in the immediate neighborhood of the lesion begin to undergo calcification. This is the resistance which the vital force offers.

It would impede the progress of caries; for the greater the density the slower is the rate of disintegration. The calcified contents, in common with the other portions, undergo decalcification as the carious portion is extended.

Portions of dentine which have been undermined, and by mechanical forces acting upon them, broken away, may be found in the cavity.

Alkalies may occasionally be the exciting cause of caries, as they have strong affinities for some of the constituents of the organic portions, and the same may be said of concentrated acids; but the removal of the inorganic portion is always accomplished by the action of acids.

Practically, the enamel is inorganic, still it contains about five per cent. of organic matter; and where this is found we will find vital force.

Destructive agents act more rapidly on the enamel than on the dentine, on account of a lesser degree of vitality. Caries attacks the cementum more rapidly than the dentine, but not so rapidly as the enamel. Caries is more common in women than in men. During pregnancy and lactation it is especially active.

Among the exciting causes of caries are defects in the enamel between the cusps, the line of union always being more or less imperfect, roughness of surface, the action of mineral or vegetable acids the sucking-bag given to children, irregularities, crowded conditions of the dental arch, artificial dentures, and eructation or emesis of acid fluids.

Among constitutional causes are scrofula, syphilis, and rachitis.

Chemical action is always the immediate cause.

Constitutional causes are concerned in producing badly organized dentures, and in producing agents acting deleteriously on the teeth. Among the different occupations most liable to produce caries, are those of dealers in pickles, cooks, and confectioners. The sugar combines with the gingival mucus, and, by fermentation, forms lactic or butyric acid, or both. Fur dealers are apt to have carious teeth, on account of the fumes of nitric acid, which is employed in coloring and cleansing furs.

In scrofula the saliva is very thick and viscid, and is tenaciously held against the necks of the teeth, acting deleteriously.

Medicinal preparations, occasionally used, which contain acids, do not necessarily cause caries, but may predispose to it. Caries is caused by an agent which, though it acts slowly, is acting constantly. It may be an excretion, a secretion, formed in the mouth, or may be introduced from without, and it acts as fast as formed.

Dental caries is divisible into three general classes: (1) Superficial, or attacking only the enamel; (2) Penetrating, or attacking the dentine; and (3) that which involves the dental pulp. The color of caries varies from white to brown or black.

White decay is most rapid, and black slowest. In the white form, all portions, both organic and inorganic, are alike acted upon. In the brown form, the inorganic parts form soluble compounds with the destructive agents, but the matrix remains untouched. In the black the salts formed are mainly insoluble, and therefore retard the progress of caries.

In chemical abrasion, soluble compounds are formed with both the organic and inorganic portions, and total removal of tooth structure takes place as far as the disease extends.

A single agent generally produces each distinct form of caries; but we may find several forms in the same mouth, or we may find a mixture of several forms in the same tooth.

Physical characteristics of caries depend on structure; but are somewhat modified by the agent producing the caries.

Chemical characteristics depend on the character of the agent. Teeth are similar in chemical composition, but not in physical structure. Poorly organized teeth offer less resistance to caries, but the resulting compounds are always the same.

Experiments on teeth out of the mouth cannot be relied upon, because we can never get the resistance which vitality offers in the mouth. Teeth are more thoroughly decalcified outside of the mouth than they could possibly otherwise be. If we burn the carious portion, we always find inorganic matter in the ash.

Teeth become carious in pairs, not from any contagion, but merely that they are the same in structure, are erupted at the same time, and are subjected to the same influences and changes.

Rocky enamel denotes a malnutrition at some portion of the time of its formation. It is found in connection with dentine, which is imperfectly formed. We find this condition in corresponding teeth on opposite sides of the jaw; and if it should be found on other teeth, it would not be at the same point, but at a point which would correspond to the development of these teeth at the time when the others were attacked.

Unless the dentine is penetrated, however, it may not be liable to caries.

The acidity of carious dentine is said to be due to the presence of superphosphate of lime. The superphosphate is formed from the phosphate by the replacement of a molecule of calcium by basic water. This changes the phosphate, which is neutral and insoluble, into a salt, which is acid and soluble. In order, however, to have removed the molecule of lime, it was necessary that an acid from an external source should previously have acted upon it. The following free acids are found in the saliva: acetic, uric, oxalic, and muriatic.

Muriatic acid is one of the most common. It is formed in the mouth by the decomposition of the chloride of sodium when an excess of salted food is eaten, or it may be formed when there is excessive inflammation of mucus membrane in the oral cavity.

Phosphate of lime is very soluble in this acid, and hence rapid destruction of inorganic matter follows its exhibition.

Galvanic action also results in its formation. Chlorate of potash, normally found in mucus, by decomposition, may produce muriatic acid.

The acids, produced by fermentation of organic matter entangled by the teeth, furnish the main portion of the destructive agent.

Mucus is ordinarily acid, and saliva alkaline, and in normal conditions one neutralizes the other.

The secretion of saliva is partially checked during fevers, and the acid mucus may then work great destruction.

Acid mucus is particularly abundant during childhood and pregnancy.

Acids first attack the enamel and roughen or erode its surface. This affords lodgment to particles of decomposing materials, which in turn decompose the tissue underneath. The character of caries depends somewhat upon the strength of the attacking acid. If diluted, it attacks primarially those portions for which it has the greatest affinity; if concentrated, it may attack all parts similarly.

Enamel protects dentine by means of its great density.

*Leptothryx buccalis* is a vegetable growth found in carious dentine, as is also often found the *oidium albicans.*

Leptothryx has the power of penetrating dental tissue, but not before the tissue has been decalcified. It is never met with in sound dentine, but is often met with in salivary calculus. Salter believes it is found in all cases of caries, and may live at the expense of the tissue.

Proximal caries occurs just above the point of contact of the teeth.

The teeth and the cornea of the eye, both being developed from skin, are liable to disease at the same time, and thus we may find keratitis and corneal opacities in conjunction with imperfectly formed dentures.

The first molars are so prone to decay, because for about six years they occupy a position similar to that of the *dens sapientiæ* in adult life. Being situated so far back, they are but imperfectly cleansed. A reason why caries is allowed to proceed so rapidly is because parents often believe them to belong to the temporary set.

Frequently as long as two years will elapse before complete eruption of the *dens sapientiæ.* During this time the gum rests upon the tooth, entangles particles of decomposing food, generates acids, and decomposition of structure results. The lower incisors are less liable to caries, because they are constantly bathed in saliva.

Salter reports a curious case:

A molar tooth was found to have three short horizontal canals piercing the neck of the tooth. On microscopic examination, the tissue in the immediate neighborhood was found to be cancellated. It extended into both crown and root, and was circumscribed. It was, therefore, true osseous structure.

The prevention of caries is in tonic constitutional treatment, thorough mastication of food, cleanliness, and the use of antacids.

The file is often beneficial in separating teeth, and the opening should be wider at the gums and larger on the lingual surfaces.

In a crowded arch, extraction of a bicuspid or molar on either side may be beneficial in spacing the teeth. To insure perfect cleanliness, floss threads should be used for passing between the teeth.

Soluble dentifrices are desirable, and should contain no gritty particles. Charcoal should never be used. Should there be a tendency to excessive acid secretion at the necks of the teeth, the mouth should be rinsed daily three or four times with *aqua calcis*, and a pinch of chalk or bicarbonate of soda rubbed occasionally on the gums is very useful.

Superficial caries may be removed by the disk or file; and if the surface is left self-cleansing, caries may be arrested.

In penetrating caries the remedy is in mechanically replacing the lost portion by some of the various preparations employed.—*Transactions Cal. State Dental Association.*

